# Effect of crosstalk between Th17 and Th9 cells on the activation of dermal vascular smooth muscle cells in systemic scleroderma and regulation of tanshinone IIA^⋆^

**DOI:** 10.1016/j.abd.2021.11.008

**Published:** 2022-09-15

**Authors:** Mengguo Liu

**Affiliations:** Department of Dermatology, Huashan Hospital, Fudan University, the 12th Urumqi Road, Shanghai, China

**Keywords:** Angiopathy, Vascular smooth muscle cells, Systemic Scleroderma, Tanshinone IIA, T-helper cell 17, T-helper cell 9, Vascular smooth

## Abstract

**Background:**

To evaluate the effect of T-helper 17 (Th17) cells and Th9 cells on the activation of dermal vascular smooth muscle cells (DVSMCs) in systemic scleroderma (SSc) and regulation of tanshinone IIA.

**Methods:**

The expression of interleukin 17 receptor (IL-17R) and interleukin 9 receptor (IL-9R) in the skin of SSc patients was assessed by immunofluorescence. The expression of IL-9 and IL-9R mRNA in peripheral blood mononuclear cells (PBMCs) of SSc patients were detected by quantitative real-time polymerase chain reaction (qRT-PCR). The proportion of Th9 cells in PBMCs of SSc patients was sorted by flow cytometry. The effect of IL-9 on the differentiation of Th17 and IL-17 on that of Th9 was detected by flow cytometry. The proportion of Th9 and Th17 cells in SSc patients was detected by flow cytometry. The level of collagen I, III, α-SMA, IL-9R, IL-17R, JNK, P38, and ERK were analyzed using western blot (WB).

**Results:**

Th9 cells were highly expressed in SSc. IL-9 stimulated the differentiation of immature T cells into Th17 cells. IL-17 induced the differentiation of immature T cells into Th9 cells. Tanshinone IIA inhibited the differentiation of immature T lymphocytes into Th17 and Th9. WB showed that the combined action of IL-17 and IL-9 upregulated the inflammation and proliferation of DVSMCs. Anti-IL17, anti-IL9, and tanshinone IIA inhibited the functional activation of DVSMCs.

**Study limitations:**

For Th17, Th9 and vascular smooth muscle cells, the study on the signal pathway of their interaction is not thorough enough. More detailed studies are needed to explore the mechanism of cell-cell interaction.

**Conclusions:**

The current results suggested that Th17 and Th9 cells induced the activation of DVSMCs in SSc through crosstalk in vitro, and tanshinone IIA inhibited the process.

## Introduction

Systemic scleroderma (SSc) is an autoimmune disease.^1^, ^2^ Autoimmune abnormality, vasculopathy, and fibroproliferative alterations are three hallmark pathological processes in SSc that are responsible for the most severe clinical manifestations of the disease. These modifications also determine the clinical outcome of the disease and mortality.^3^ Vasculopathy and fibrosis occur in the early stages and exist throughout the course of the disease and are regulated by autoimmunity. Vasculopathy is characterized by vascular endothelial cell inflammation and smooth muscle cell proliferation, which eventually leads to vascular fibrosis and stenosis.^4^, ^5^, ^6^ However, the pathogenesis of SSc-related vasculopathy is not yet understood.

Recent studies have shown an increase in T-helper 17 (Th17) cells and **IL-17** in the skin and peripheral blood of SSc patients.^7^ Interleukin-17A (IL-17A) induces inflammation of vascular endothelial cells.^8^ The authors also observed that the proliferation and migration of dermal vascular smooth muscle cells (DVSMCs) from media to intima induced by Th17 cells as well as the phenotype transformation from contraction type to synthetic type, which results in over-production of collagen, may play a key role in the pathogenesis of SSc.^9^, ^10^, ^11^ However, whether Th17 cells and other lymphocytes act synergistically in inducing the function of DVSMCs is yet to be elucidated. Recently, Interleukin-9 (IL-9) and IL-9-producing CD4^+^ T cells (T-helper 9 (Th9) cells) have been found in SSc, suggesting that the IL-9 axis might be involved in SSc pathogenesis.^12^, ^13^ Other studies have shown that IL-9 induces the differentiation of Th17 cells.^14^, ^15^ Nevertheless, none of the studies have specifically addressed whether Th17 and Th9 cells interact with each other to promote the functional activation of DVSMCs in the pathogenesis of SSc.

Thus, the present study elaborated on the effect of Th17 and Th9 cells on SSc patient-derived Dermal Vascular Smooth Muscle Cells (DVSMCs) The authors also studied the regulatory effect of tanshinone IIA on this process. Interestingly, the authors observed that Th17 and Th9 cells might induce the activation of DVSMCs in vitro in SSc through “dialogue”, and tanshinone IIA alleviates this effect. Tanshinone IIA is a fat-soluble pharmacologically active component of the Chinese herb Salvia miltiorrhiza, a well-known traditional Chinese medicine that is used to treat cardiovascular diseases and connective tissue diseases, such as systemic lupus erythematosus, scleroderma, and rheumatoid arthritis. Numerous research has demonstrated that tanshinone IIA inhibits the proliferation and migration of artery smooth muscle cells, reduces pulmonary artery pressure, and ameliorates hypoxia-induced pulmonary artery remodeling. Meanwhile, the authors’ previous results provide preliminary evidence that tanshinone IIA exerts an inhibitory effect on IL-17A-induced proliferation, collagen synthesis, and migration of SSc patient-derived DVSMCs. These findings potentially explain why Salvia miltiorrhiza clearly relieves symptoms in SSc patients in the clinic.

## Methods

### SSc patients and healthy controls

A total of 10 patients with SSc (4 men and 6 women) were included in this study after they provided informed consent. All patients were diagnosed according to the 2013 classification criteria for SSc and presented different degrees of clinical manifestations of microangiopathy (for example, Raynaud phenomenon, digital ulcers, telangiectasia, pulmonary hypertension, and renal crisis). In addition, 10 age- and sex-matched healthy individuals (5 men and 5 women), who underwent surgery for non-cutaneous disease evaluation, were enrolled in this study. Blood samples were withdrawn from SSc patients and healthy individuals. The skin tissues of SSc patients were obtained to isolate DVSMCs.

### Isolation of SSc patient-derived DVSMCs

The skin tissues of the lesions were disinfected using 75% alcohol and excised surgically under sterile conditions, followed by 6–8 washes in sterile phosphate-buffered saline (PBS) and then soaked in 75% alcohol for 5–10 min. Subsequently, the tissues were washed with PBS containing two types of antibiotics (penicillin and streptomycin). The dermal vascular tissue was isolated under a dissecting microscope, minced, and trypsinized. The undigested tissue was removed using a 200-mesh filter and centrifugation for 10 min at 1500 rpm. Finally, the cells obtained were resuspended and added to the smooth muscle cell basal medium (Promocell Heidelberg, Germany).

### Tissue immunofluorescence

The skin tissue of SSc patients was trimmed, dehydrated, embedded, sliced, baked, dewaxed, and rehydrated. Skin tissue slices were blocked with 1% bovine serum albumin (BSA) for 30 min and incubated with primary antibody overnight at 4 °C. After incubation with the secondary antibody at room temperature for 1 h, the cell nuclei were stained with 49,6-Diamidino-2-Phenylindole (DAPI) was used to stain the cell nuclei. The images were scanned using a fluorescence microscope (Olympus, Tokyo, Japan).

### Cell immunofluorescence

DVSMCs were plated in 24-well plates with coverslips. At 90%–100% confluency, the cells were stimulated for 24 h, followed by fixation with 4% paraformaldehyde for 15 min and permeabilization with 0.5% Triton X-100 for 15 min. Then, the cells were blocked with 1% BSA for 30 min and incubated with primary antibody overnight at 4 °C. After incubation with a secondary antibody at room temperature for 1 h, DAPI was used to stain the cell nuclei. The images were scanned using a fluorescence microscope.

### Enzyme-linked immunosorbent assay (ELISA)

The concentration of IL-9 was detected using the ELISA kit (MultiSciences, Hangzhou, China) according to the manufacturer’s instructions. An equivalent of 100 μL standard protein samples and cell culture supernatants were added and incubated for 2 h at room temperature. After washing 4–6 times, each well was incubated for 1 h with 50 μL primary IL-9 antibody and then with 100 μL of enzyme-labeled antibody for 1 h at room temperature. After the termination of the reaction, the OD was measured at 450 nm maximum absorption and 630 nm wavelength. The calibrated OD value was obtained by subtracting the 630 nm value from that of 450 nm. The IL-9 concentration data were presented as mean ± SD values.

### Real-time RT-PCR

Total RNA of DVSMCs was extracted using TRIzol reagent. Complementary DNA (cDNA) samples were synthesized using the First Strand cDNA Synthesis Kit (Yeasen, Shanghai, China) and oligo (dT) primers. The levels of mRNA of the target genes were examined using SYBR Green PCR Master Mix. The following primer pairs were used:

IL-9

Forward: TCA AGATGCTTCTGGCCATG

Reverse: AGGGAATGCCCAAACAGAGA

IL-9R

Forward: CCAGCACAGGGATCACATTG

Reverse: GCCTGTATAACGCTCCTCCT

*β-actin*

Forward: AACCATGAGGGAAATCGTGC

Reverse: CAGGATGGCACGAGGAACAT

The 2^−ΔΔCt^ method was used to normalize the transcription to the level of β-actin and to calculate the fold-induction relative to the controls.

### Peripheral blood mononuclear cells (PBMCs) and CD4^+^ T-cells sorting and flow cytometry detection

PBMCs were obtained by density gradient centrifugation using Ficoll-Hypaque and were cultured in RPMI 1640 medium supplemented with 10% FCS and antibiotics from SSc patients and healthy controls. CD4^+^ T-cell subset was sorted using Magnetic-Activated Cell Sorting (MACS). The induced cells were incubated with FITC-labeled anti-human CD4 antibody at 4 °C for 30 min. In order to further analyze the production of IL-9 or IL-17, the cells were permeated with permeable buffer and stained with PE-conjugated anti-human IL-9 or anti-human IL-17 at room temperature for 30 min. At the end of the staining, all stained cells were analyzed by flow cytometry.

### Western blot

The proteins of DVSMCs were extracted using RIPA lysis buffer (Beyotime, Shanghai, China) supplemented with the protease inhibitor Phenylmethanesulfonylfluoride Fluoride (PMSF). The proteins were separated by 8% SDS-PAGE and transferred onto PVDF membranes. Subsequently, the membranes were blocked with 5% milk for 2 h at room temperature, and probed with primary antibodies, including collagen I (1:2000, Abcam), collagen III (1:5000, Abcam), α-SMA (1:1000, CST), p38 (1:1000, CST), phospho-p38 (1:1000, CST), ERK (1:2000, CST), phospho-ERK (1:1000, CST), and GAPDH (1:5000, Proteintech) overnight at 4 °C, and then incubated with appropriate Horseradish Peroxidase (HRP) conjugated secondary antibodies for 1.5 h at room temperature. The proteins were detected using ECL detection reagents.

### Statistical analysis

The data were statistically analyzed using the SPSS version 20.0 software (SPSS Inc., Chicago, IL, USA). Quantitative data are expressed as the means ± Standard Deviation (SD). Data between the patients and healthy individual groups were analyzed using Student’s *t*-test. Data among multiple groups were analyzed using one-way analysis of variance (ANOVA) combined with Bonferroni correction; p-value < 0.05 was statistically significant.

## Results

### Correlation between Th9 and SSc

**Figure 1:**
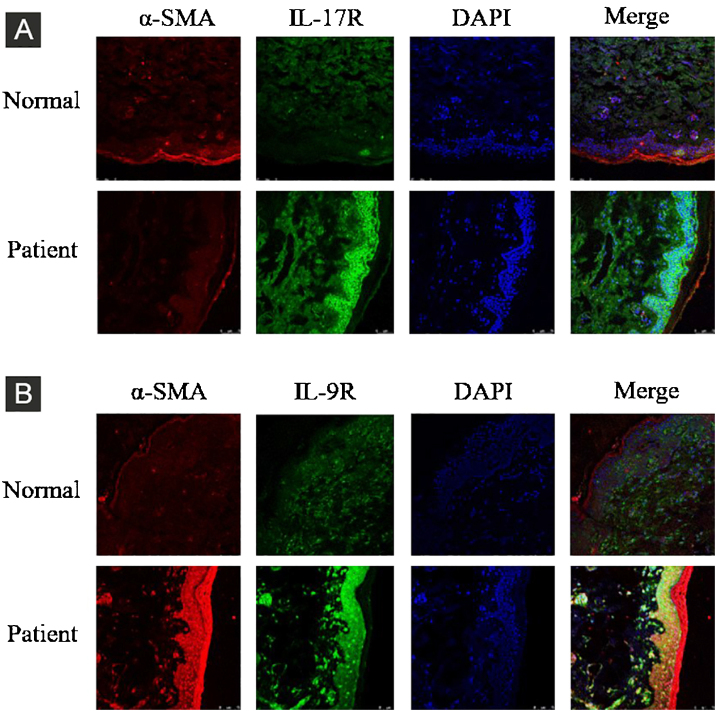
IL-17R and IL-9R were highly expressed in skin tissues of SSc patients. A, Compared to healthy control, IL-17R was highly expressed in the skin tissue of SSc patients. α-SMA, red fluorescence, 1:200. IL-17R, green fluorescence, 1:200. B, Compared to healthy control, IL-9R was highly expressed in the skin tissue of SSc patients. α-SMA, red fluorescence, 1:200. IL-9R, green fluorescence, 1:200.

**Figure 2:**
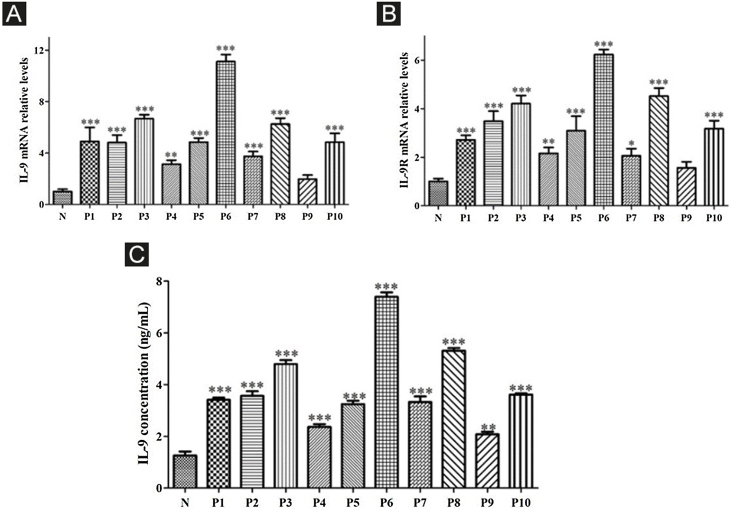
High expression of IL-9 and IL-9R in patients with SSc. A, High expression of IL-9 mRNA in PBMCs of SSc patients. B, High expression of IL-9R mRNA in PBMCs of SSc patients. C, High expression of IL-9 in the serum of SSc patients. N means Normal, P means Patient. * vs. N, p < 0.05. ** vs. N, p < 0.01. *** vs. N, p < 0.001.

**Figure 3:**
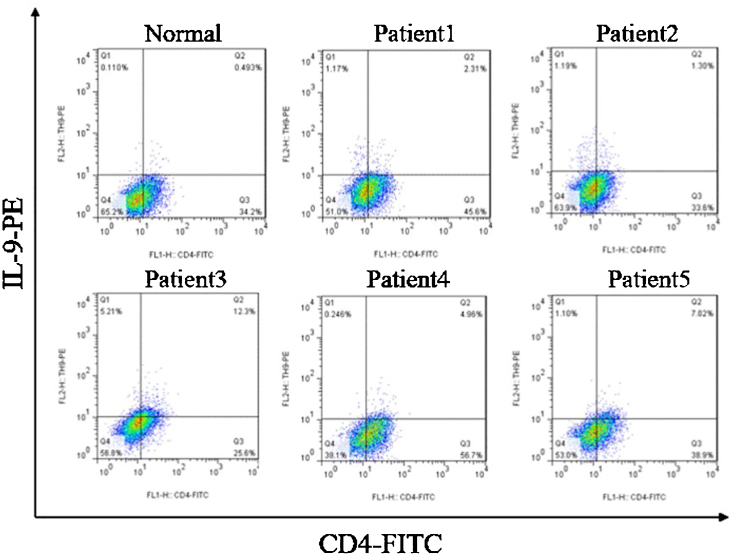
The proportion of CD4^+^IL-9^+^ T-cells in PBMCs of SSc patients was significantly higher than that of healthy controls.

Compared to normal subjects, IL-9R and IL-17R were highly expressed in the skin lesions of SSc patients (Fig. 1). IL-9 and IL-9R mRNA levels of PBMCs and IL-9 levels in SSc serum were increased (Fig. 2). Flow cytometry showed that the proportion of CD4^+^ IL-9^+^ T cells in PBMCs of SSc patients was significantly higher (Fig. 3). The proportion of Th9 was associated with SSc.

### Crosstalk between Th17 and Th9 cells in vitro and regulation of tanshinone IIA

#### IL-9 and SSc serum promote Th17 differentiation and the reversal effect of tanshinone IIA

**Figure 4:**
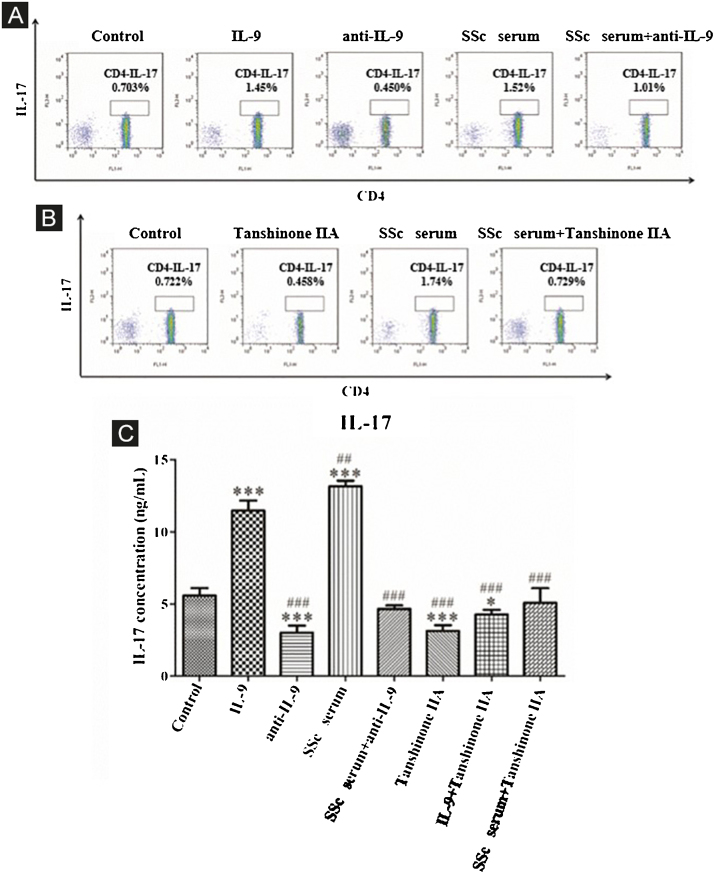
IL-9 neutralizing antibody and Tanshinone IIA inhibit the differentiation of immature T-lymphocytes into Th17. (A and B) The proportion of immature T-lymphocytes differentiated into Th17 in each group was detected using flow cytometry. (C) The content of IL-17 secreted by immature T lymphocytes in each group was detected by ELISA. IL-9 concentration 20 ng/mL, anti-IL-9 concentration 5 μg/mL, Tanshinone IIA concentration 50 μM. * vs. control group, p < 0.05, *** vs. control group, p < 0.001, ^##^ vs. IL-9 group, p < 0.01, ^###^ vs. IL-9 group, p < 0.001.

The results showed that the proportion of Th17 cells in IL-9 and SSc serum groups was significantly higher than that in the control group; however, the proportion of Th17 cells in the IL-9 neutralizing antibody group was significantly lower than that in the control group, and IL-9 neutralizing antibody decreased the promoting effect of serum on Th17 cells (Fig. 4A). These findings indicated that IL-9 stimulates the differentiation of immature T-lymphocytes into Th17 cells in vitro, as well as the differentiation of immature T-lymphocytes into Th17 cells. The data showed that tanshinone IIA decreases the proportion of Th17 and inhibits the effect of SSc serum on Th17 cells as compared to the control (Fig. 4B). Moreover, the content of IL-17 in the culture supernatant of each group was detected by ELISA. These results showed that IL-9 and SSc serum significantly promotes the secretion of IL-17 by immature T-lymphocytes, while IL-9 neutralizing antibody and tanshinone IIA significantly inhibit the secretion of IL-17 by immature T-lymphocytes and reverse the triggering effect of IL-9 and SSc serum (Fig. 4C).

#### IL-17 and SSc serum promote Th9 differentiation and the reversal effect of tanshinone IIA

**Figure 5:**
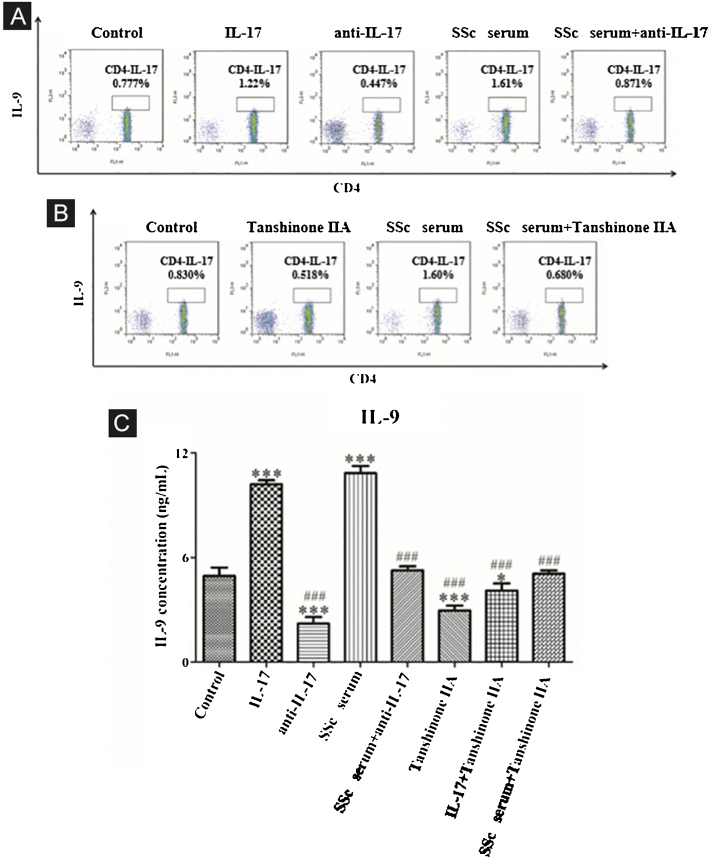
IL-17 neutralizing antibody and Tanshinone IIA inhibit the differentiation of immature T-lymphocytes into Th9. (A and B) The proportion of immature T-lymphocytes differentiated into Th9 in each group was detected using flow cytometry. (C). The content of IL-9 secreted by immature T-lymphocytes in each group was detected by ELISA. IL-17 concentration 100 ng/mL, anti-IL-17 concentration 8 μg/mL, Tanshinone IIA concentration 50 μM. * vs. control group, p < 0.05, *** vs. control group, p < 0.001, ^###^ vs. IL-9 group, p < 0.001.

The results showed that the proportion of Th9 cells in the IL-17 and SSc serum groups was significantly higher than that in the control group; however, the proportion of Th9 cells in the IL-17 neutralizing antibody group was significantly lower than that in the control group and IL-17 neutralizing antibody decreased the promoting effect of serum on Th9 cells (Fig. 5A). These findings indicated that IL-17 stimulates the differentiation of immature T-lymphocytes into Th9 cells in vitro, and IL-17 in the serum of SSc patients stimulates the differentiation of immature T-lymphocytes into Th9 cells. In addition, tanshinone IIA decreases the proportion of Th9 and inhibits the promoting effect of SSc serum on Th9 cells as compared to the control (Fig. 5B). Moreover, the content of IL-9 in the culture supernatant of each group was detected by ELISA. The results showed that IL-17 and SSc serum significantly promoted the secretion of IL-9 by immature T lymphocytes, while IL-17 neutralizing antibody and tanshinone IIA significantly inhibited the secretion of IL-9 by immature T-lymphocytes and reversed the promoting effect of IL-17 and SSc serum (Fig. 5C).

### Effects of IL-17 and IL-9 on the functional activation of DVSMCs and regulation of tanshinone IIA

#### IL-9 and SSc serum promote the expression of IL-17R in DVSMCs, and tanshinone IIA reverses this effect

**Figure 6:**
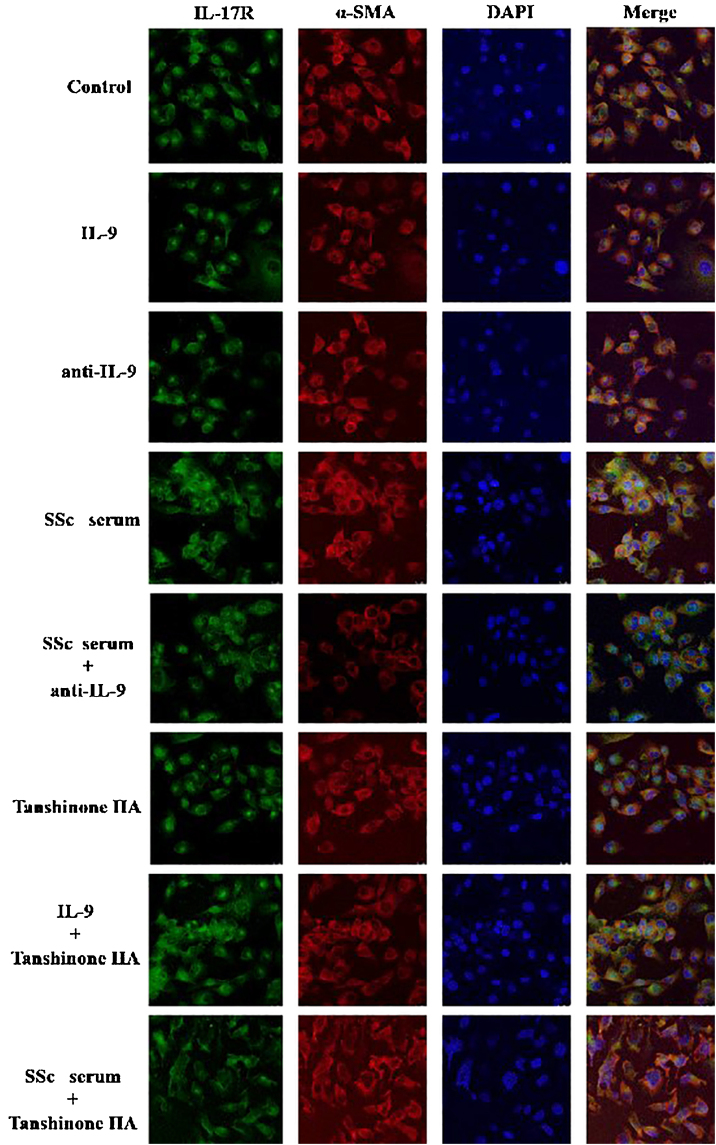
IL-9 neutralizing antibody and Tanshinone IIA inhibit the expression of IL-17R in DVSMCs. α-SMA, red fluorescence; IL-17R, green fluorescence.

The DVSMCs of SSc patients were isolated and incubated with IL-9, IL-9 neutralizing antibody, SSc serum, SSc serum and IL-9 neutralizing antibody, tanshinone IIA, IL-9, and tanshinone IIA, and SSc serum and tanshinone IIA for 3 days. The expression of IL-17R was detected by immunofluorescence. The results showed that IL-9 and SSc serum significantly promote the expression of IL-17R, and IL-9 neutralizing antibody and tanshinone IIA reversed the promoting effect of IL-9 and SSc serum (Fig. 6).

#### IL-17 and SSc serum promote the expression of IL-9R in DVSMCs, and tanshinone IIA reverses this effect

**Figure 7:**
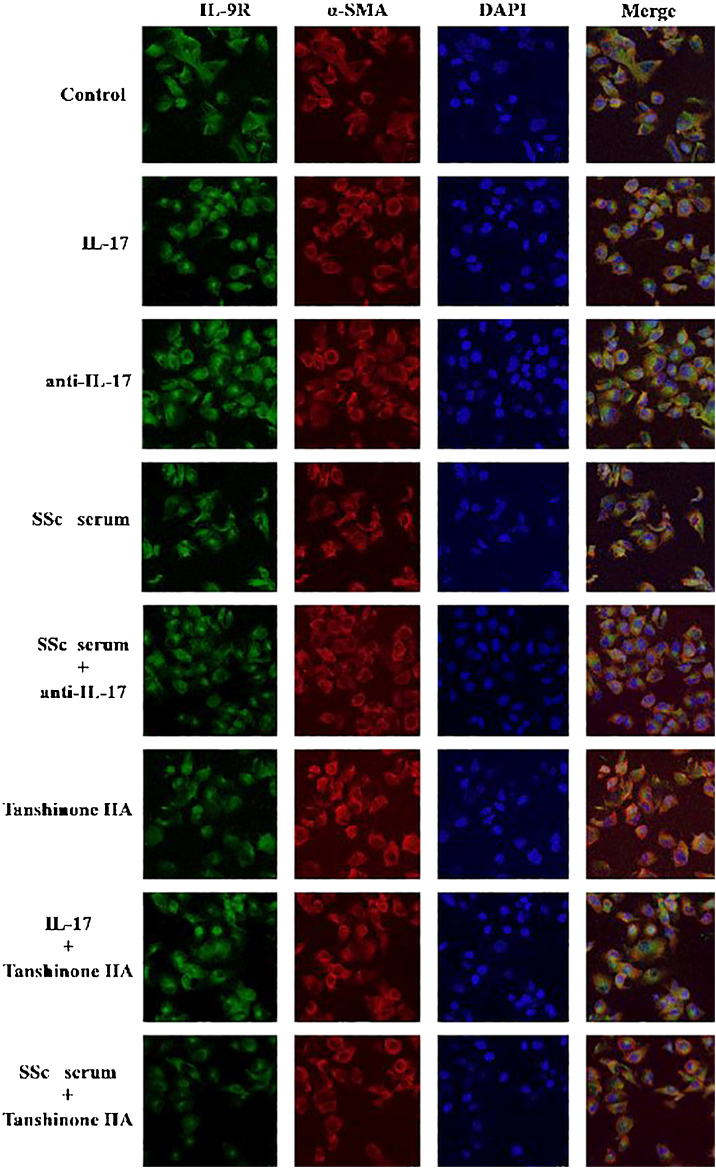
IL-17 neutralizing antibody and Tanshinone IIA inhibit the expression of IL-9R in DVSMCs. α-SMA, red fluorescence; IL-9R, green fluorescence.

The DVSMCs were incubated with IL-17, IL-17 neutralizing antibody, SSc serum, SSc serum, and IL-17 neutralizing antibody, tanshinone IIA, IL-17, and tanshinone IIA, and SSc serum and tanshinone IIA for 3 days. The expression of IL-9R was detected by immunofluorescence. The results showed that IL-17 and SSc serum significantly promote the expression of IL-9R, and IL-17 neutralizing antibody and tanshinone IIA reversed the promoting effect of IL-17 and SSc serum (Fig. 7).

#### IL-9 and SSc serum promote the functional activation of DVSMCs, and tanshinone IIA reverses this effect

**Figure 8:**
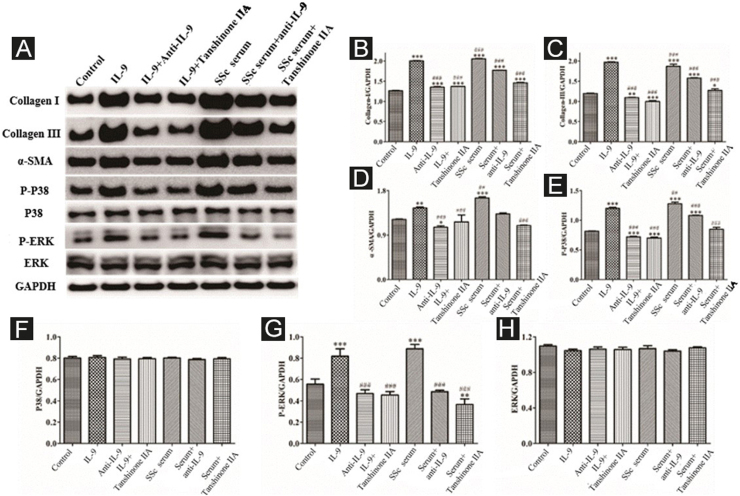
Expression of various fibrosis-related and signaling pathway-related proteins in functionally-activated DVSMCs were detected by Western blot. A, mmunoreactive bands of Western blot of various proteins. IL-9 promotes the expression of various proteins in DVSMCs, which is inhibited by Tanshinone IIA. (B–H) Western blot data of collagen I, collagen III, α-SMA, P-P38, P-ERK, and ERK are represented as statistical graphs.

The DVSMCs were incubated with IL-9, IL-9, and IL-9 neutralizing antibodies, IL-9 and tanshinone IIA, SSc serum, SSc serum and IL-9 neutralizing antibodies, and SSc serum and tanshinone IIA for 3 days. Collagen I, collagen III, α-SMA, P-P38, P38, P-ERK, and ERK were detected by WB. The results showed that IL-9 and SSc serum significantly promote the expression of collagen I, collagen III, α-SMA, P-P38, and P-ERK. Moreover, IL-9 neutralizing antibody and tanshinone IIA reversed the effects of IL-9 and SSc serum (Fig. 8).

#### IL-17 and SSc serum promote the functional activation of DVSMCs, and tanshinone IIA reverses this effect

**Figure 9:**
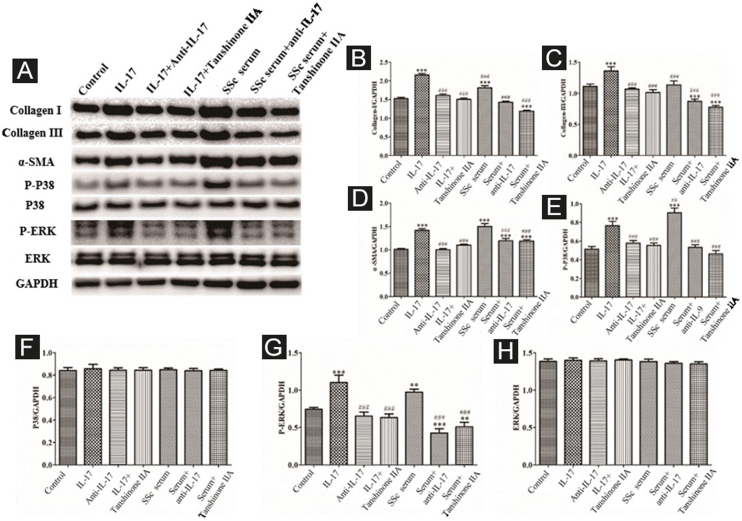
Expression of various fibrosis-related and signaling pathway-related proteins in functionally-activated DVSMCs were detected by Western blot. A, Immunoreactive bands of Western blot of various proteins. IL-17 promotes the expression of various proteins in DVSMCs, which is inhibited by Tanshinone IIA. (B–H) Western blot results of collagen I, collagen III, α-SMA, P-P38, P-ERK, and ERK are expressed in the form of statistical graphs.

The DVSMCs were incubated with IL-17, IL-17, and IL-17 neutralizing antibodies, IL-17 and tanshinone IIA, SSc serum, SSc serum, and IL-17 neutralizing antibodies, and SSc serum and tanshinone IIA for 3 days. Collagen I, collagen III, α-SMA, P-P38, P38, P-ERK, and ERK were detected by WB. The results showed that IL-17 and SSc serum significantly promote the expression of collagen I, collagen III, α-SMA, P-P38, and P-ERK. IL-17 neutralizing antibody and tanshinone IIA reversed the effects of IL-17 and SSc serum (Fig. 9).

#### Tanshinone IIA inhibits the synergistic effect of IL-9 and IL-17 on functional activation of DVSMCs

**Figure 10:**
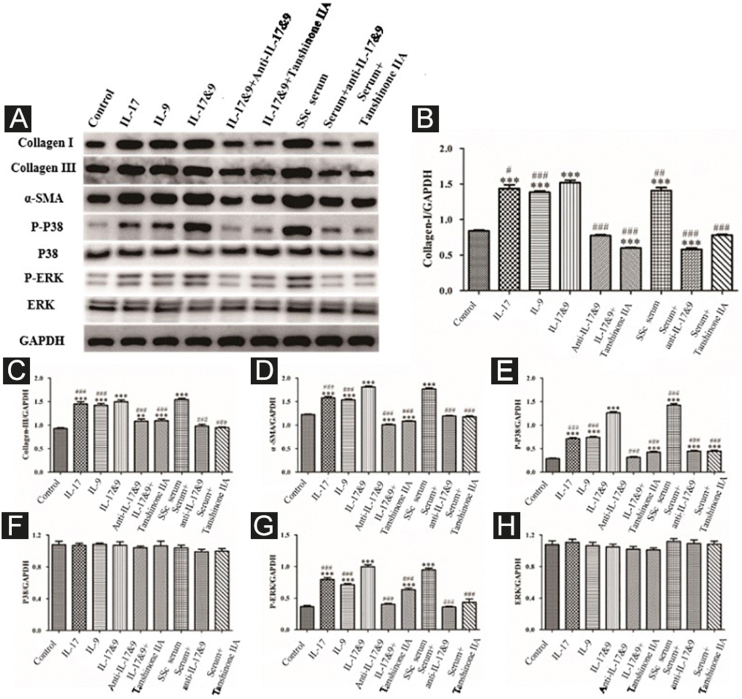
Expression of various fibrosis-related and signaling pathway-related proteins in functional activated DVSMCs were detected by Western blot. A, Immunoreactive bands of Western blot of various proteins. IL-9 and IL-17 play a synergistic role in promoting the expression of various proteins in DVSMCs, which is inhibited by Tanshinone IIA. (B–H) Western blot data of collagen I, collagen III, α-SMA, P-P38, P-ERK, and ERK are represented as graphs.

DVSMCs were treated with IL-17, IL-9, IL-17, and IL-9, IL-17 neutralizing antibody and IL-9 neutralizing antibody, IL-17 and IL-9 and tanshinone IIA, SSc serum, SSc serum/IL-17 neutralizing antibody/IL-9 neutralizing antibody, and SSc serum and tanshinone IIA for 3 days. The proteins were collected for WB detection of collagen I, collagen III, α-SMA, P-P38, P38, P-ERK, and ERK. The results showed that IL-17, IL-9, and SSc serum significantly promote the expression of collagen I, collagen III, α-SMA, P-P38, and P-ERK. In addition, IL-9 and IL-17 neutralizing antibodies and tanshinone IIA inhibited the synergistic effect of IL-9 and IL-17 (Fig. 10).

## Discussion

Th9, a defined subset of T-helper cells, has been identified by the potent production of IL-9, which is a protein of 144 amino acids with a secretory signal sequence of 18 amino acids.^16^ Interestingly, IL-9 binds to IL-9R on target cells.^17^ Although the role of the Th9 cell subset has been described recently in many types of inflammatory diseases (namely, atopic diseases, parasitic inflammation, experimental autoimmune encephalomyelitis, and inflammatory bowel diseases),^18^, ^19^, ^20^, ^21^, ^22^, ^23^ the role in the pathogenesis of rheumatic diseases, such as SSc, is yet unknown.

The presence of high IL-9 levels and the strong expression of Th9 in SSc raise the concern of whether Th9 cells might participate in SSc pathogenesis. In this study, the strong expression of IL-9R has been demonstrated recently in the skin tissue of patients with SSc. Also, IL-9 expression was observed in the PBMCs from SSc patients. The majority of IL-9-producing cells in the skin were identified as Th9 cells. Among peripheral blood mononuclear cells, Th9 cells are the major source of IL-9, which is significantly expanded in SSc patients as compared to the controls. The participation of IL-9 and Th9 in the process of inflammation and fibrosis in SSc is yet elucidated.

The previous research has confirmed that IL-17 and Th17 enhance the proliferation, collagen synthesis and secretion, and migration of SSc patient-derived DVSMCs via the Mitogen-Activated Protein Kinase (MAPKs) signaling pathway.^9^ In addition, the previous study also demonstrated that tanshinone IIA (C19H18O3), a fat-soluble pharmacologically active component of the Chinese herb *Sativa miltiorrhiza* is a well-known traditional Chinese medicine used for the treatment of cardiovascular and connective tissue diseases, exerts an inhibitory effect on the functional activation of SSc patient-derived DVSMCs.^10^

The present study focuses on the co-effects of Th17 and Th9 cells on SSc patient-derived DVSMCs. The authors also studied the regulatory effect of tanshinone IIA on this process. Th17 and Th9 promote each other’s differentiation from immature T lymphocytes via characteristic cytokines IL-17 and IL-9. IL-17, IL-9, and SSc serum promoted the expression of collagen I, collagen III, α-SMA, P-P38, and P-ERK, inducing the functional activation of DVSMCs. Increased collagen synthesis and secretion of DVSMCs indicates the aggravation of angiopathy and fibrosis in SSc. Nevertheless, IL-17 and IL-9 neutralizing antibodies and tanshinone IIA reverse the effects.

Several aspects of research evidence have demonstrated the close association of Th9 with Th17 cell development. First, IL-9 could induce the differentiation of naïve CD4^+^ T cells into Th17 cells. Second, IL-9 amplifies Th17 development in a positive autocrine loop. Furthermore, IL-9 might be involved in the development of autoimmune disease through Th17-associated inflammation and vasculopathy. Consistent with the relationship between IL-9 and Th17 cell development, a positive correlation of Th9 cells with Th17 cells as well as IL-9 with IL-17 was observed in SSc patients, indicating Th9/IL-9 and Th17/IL-17 might be cooperatively involved in the pathogenesis of SSc. However, the exact mechanism by which Th9 and Th17 regulate each other during the development of SSc remains unclear and requires further investigation.

## Conclusions

The results of this study revealed the effect of T-cell interaction on the functional activation of SSc VSMCs. Thus, the correlation between cellular immune abnormality and angiogenesis in the pathogenesis of SSc is detected, which provides a theoretical and experimental basis for SSc to find new therapeutic targets and drugs.

## Financial support

This work was supported by the National Natural Science Foundation of China (81602747).

## Author’s contributions

Mengguo Liu designed and conducted the experiments, performed the statistical analysis, and wrote and revised the manuscript.

## Conflict of interest

None declared.
